# Opening the Black Box of an mHealth Patient-Reported Outcome Tool for Diabetes Self-Management: Interview Study Among Patients With Type 2 Diabetes

**DOI:** 10.2196/47811

**Published:** 2023-09-19

**Authors:** Christina Marini, Jocelyn Cruz, Leydi Payano, Ronaldo Patino Flores, Gina-Maria Arena, Soumik Mandal, Eric Leven, Devin Mann, Antoinette Schoenthaler

**Affiliations:** 1 Department of Neurology, NYU Langone New York, NY United States; 2 Center for Healthful Behavior Change, Institute for Excellence in Health Equity, NYU Langone Health New York, NY United States; 3 Department of Technology Management & Innovation, NYU Tandon School of Engineering New York, NY United States; 4 Department of Population Health, Healthcare Innovation Bridging Research, Informatics and Design Lab, NYU Langone Health New York, NY United States

**Keywords:** mobile health, mHealth, patient-reported outcomes, diabetes, qualitative, patient engagement

## Abstract

**Background:**

Mobile health (mHealth) tools are used to collect data on patient-reported outcomes (PROs) and facilitate the assessment of patients’ self-management behaviors outside the clinic environment. Despite the high availability of mHealth diabetes tools, there is a lack of understanding regarding the underlying reasons why these mHealth PRO tools succeed or fail in terms of changing patients’ self-management behaviors.

**Objective:**

This study aims to identify the factors that drive engagement with an mHealth PRO tool and facilitate patients’ adoption of self-management behaviors, as well as elicit suggestions for improvement.

**Methods:**

This qualitative study was conducted within the context of a randomized controlled trial designed to evaluate the efficacy of an mHealth PRO tool (known as i-Matter) versus usual care regarding reduction in glycated hemoglobin (HbA_1c_) levels and adherence to self-management behaviors at 12 months among patients with uncontrolled type 2 diabetes. Patients randomized to i-Matter participated in semistructured interviews about their experiences at the 3-, 6-, 9-, and 12-month study visits. A qualitative analysis of the interviews was conducted by 2 experienced qualitative researchers using conventional qualitative content analysis.

**Results:**

The sample comprised 71 patients, of whom 67 (94%) completed at least one interview (n=48, 72% female patients; n=25, 37% identified as African American or Black; mean age 56.65 [SD 9.79] years). We identified 4 overarching themes and 6 subthemes. Theme 1 showed that the patients’ reasons for engagement with i-Matter were multifactorial. Patients were driven by internal motivating factors that bolstered their engagement and helped them feel accountable for their diabetes (subtheme 1) and external motivating factors that helped to serve as reminders to be consistent with their self-management behaviors (subtheme 2). Theme 2 revealed that the use of i-Matter changed patients’ attitudes toward their disease and their health behaviors in 2 ways: patients developed more positive attitudes about their condition and their ability to effectively self-manage it (subtheme 3), and they also developed a better awareness of their current behaviors, which motivated them to adopt healthier lifestyle behaviors (subtheme 4). Theme 3 showed that patients felt more committed to their health as a result of using i-Matter. Theme 4 highlighted the limitations of i-Matter, which included its technical design (subtheme 5) and the need for more resources to support the PRO data collected and shared through the tool (subtheme 6).

**Conclusions:**

This study isolated internal and external factors that prompted patients to change their views about their diabetes, become more engaged with the intervention and their health, and adopt healthy behaviors. These behavioral mechanisms provide important insights to drive future development of mHealth interventions that could lead to sustained behavior change.

## Introduction

### Background

Diabetes is a nationwide epidemic that affects >37 million adults in the United States, with 1.4 million new cases diagnosed each year [1]. The growing prevalence of diabetes is associated with an estimated US $327 billion in lost work, wages, and medical costs [2]. Despite the availability of evidence-based guidelines for effective diabetes care [3],  downward trends in glycemic control (glycated hemoglobin [HbA_1c_] level <7%: 57.4% in 1999 vs 50.5% in 2018) suggest that the burden of diabetes will only worsen [4]. On a daily basis, patients are required to make multiple diabetes self-management decisions such as taking medications, making food choices, and engaging in physical activity outside the clinic environment, all of which have great impact on their health [5]. Nevertheless, most of the focus of diabetes care has been on evaluating care through the lens of clinical parameters such as HbA_1c_ levels and changes in micro- and macrovascular complications and less on patients’ perspective of diabetes self-management [6]. To address the growing burden of diabetes, the measures of disease improvement must encapsulate the day-to-day impact of diabetes on patients’ lives.

Patient-reported outcomes (PROs) are a standardized and quantifiable measurement approach that facilitates the collection and integration of data on patients’ perspectives into the clinical management of diabetes [7]. A growing number of studies are using mobile health (mHealth) platforms that enable real-time collection of PROs to facilitate the assessment of patient self-management behaviors outside the clinic environment, enhance patient engagement in their care, and inform clinical decision-making, demonstrating positive short-term benefits on clinical and behavioral outcomes [8-10]. To date, there are >200 commercially available mHealth apps that capture and visualize patient-reported data for diabetes (eg, Glucose Buddy and BlueStar) [11-13].

### Objectives

mHealth interventions can benefit patients’ diabetes care behaviors by facilitating individualized and integrated care that promotes self-management behaviors and enhances diabetes quality of life [14]; for example, Lauffenburger et al [15] demonstrated that positively framed SMS text messaging had a significant impact on engagement with the intervention (eg, higher response rate) of individuals with type 2 diabetes, which ultimately has an effect on behavior change. However, a recent analysis of 17 systematic reviews of mHealth interventions for type 2 diabetes found mixed evidence for the effect of SMS text messaging on diabetes self-management behaviors and reduction in HbA_1c_ levels [16]. Although the heterogeneity of the included studies made it difficult to determine the causes of the mixed findings, a cross-cutting limitation of the included studies was the limited insights into the underlying reasons why these mHealth interventions succeed or fail in terms of changing patients’ self-management behaviors [16]. To fill this gap, this qualitative study explored patients’ perspectives of an mHealth PRO tool for diabetes management within the context of an ongoing randomized controlled trial with the goals of (1) identifying the factors that drive engagement with an mHealth PRO tool and facilitate patients’ adoption of self-management behaviors and (2) eliciting suggestions for improvement.

## Methods

### Overview of i-Matter

**Figure 1:**
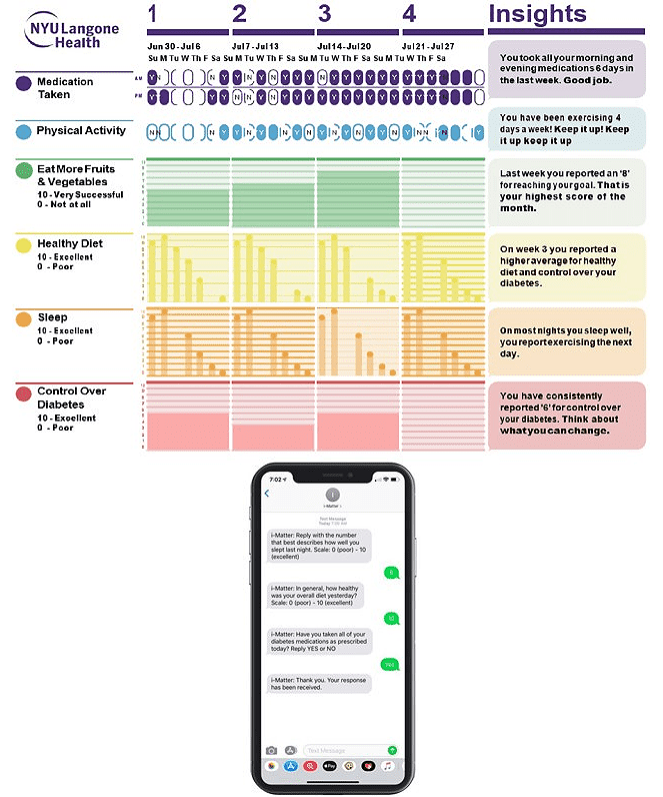
Patient-facing i-Matter features.

We are currently conducting a randomized controlled trial to evaluate the efficacy of an mHealth PRO tool (intervention) versus usual care regarding reduction in HbA_1c_ levels and adherence to self-management behaviors at 12 months among 282 patients with uncontrolled type 2 diabetes who are receiving care in primary care practices. The mHealth PRO tool, known as i-Matter, is a theoretically grounded technology solution informed by the capability, opportunity, motivation, and behavior model, which posits that for any behavior to occur, a person must have the capability, opportunity, and motivation to perform the behavior. i-Matter comprises three key features (Figure 1): (1) SMS text messages that capture patients’ self-reported PROs in real time, (2) data-driven feedback and motivational SMS text messages based on responses to the PROs, and (3) dynamic visualizations of the PROs that are shared in personalized PDF reports and integrated into the clinic electronic health record (EHR) [16].

Over the 12-month study, patients receive up to 5 daily PRO SMS text messages that assess their sleep quality, physical activity, diet, and medication adherence. PRO messages are sent in the morning (sleep, diet, and medication adherence) and evening (physical activity and an optional second medication adherence message). The messages require patients to respond with either a yes or no response or a response on a scale ranging from 0 to 10 within a 2-hour period. Patients also receive 2 weekly messages in the afternoon that assess their diabetes, quality of life, and attainment of a personalized health goal. Personalized PDF reports are sent to patients monthly and visualize patients’ responses to the PRO messages over time. Details regarding the development of i-Matter are reported elsewhere [17]. After consent and baseline procedures, patients are randomized to i-Matter or usual care in a 1:1 ratio using an SAS (SAS Institute Inc) macro developed by the blinded study statistician. This paper focuses on the patients randomized to i-Matter who completed the trial and participated in interviews at the 3-, 6-, 9-, and 12-month study visits.

### Patient Recruitment Into the Trial

Patients are being recruited for the trial from a network of primary care practices of NYU Langone Health across the 5 boroughs of New York City and Long Island, New York. Details of our recruitment approach are reported elsewhere [17]. Briefly, to participate in the trial, patients must (1) have a diagnosis of type 2 diabetes for ≥6 months, (2) have uncontrolled type 2 diabetes defined as HbA_1c_ level >7% documented in the EHR at least twice in the past year, (3) be fluent in English or Spanish, (4) be willing to send and receive SMS text messages, and (5) be aged ≥18 years. Patients are excluded if they (1) refuse or are unable to provide informed consent; (2) have acute renal failure, end-stage renal disease or evidence of dialysis, renal transplantation, or other end-stage renal disease–related services documented in the EHR; (3) are participating in another diabetes study; (4) have significant psychiatric comorbidity or reports of substance abuse (as documented in the EHR); (5) are pregnant or planning to become pregnant within 12 months; or (6) plan to discontinue care at the practice within the next 12 months.

Study participants were given oral and written descriptions of the research and the benefits and risks, as well as their rights, and information on the study team or principal investigator by the research team, along with answers to any of their questions. Participants were informed that they could retract their responses or participation at any time and that their participation or refusal to participate would not affect their care.

### Ethics Approval

The NYU Langone Health Institutional Review Board approved the study (i18-01044).

### Patient Interviews

Patients who were randomized to i-Matter participated in semistructured individual interviews about their experiences at the 3-, 6-, 9-, and 12-month study visits. The interviews were conducted after the completion of the validated outcome measures that assessed adherence to diabetes self-management behaviors (eg, medication adherence, diabetes diet, and foot care), diabetes self-efficacy, and patient-physician communication [16]. The interviews at each time point were brief and designed to help the study team understand the behavior change process and engagement with our intervention over time. A major shortcoming of most mHealth studies is the failure to include qualitative measures that elucidate why their digital tool may or may not be working in the target population [17]. The use of multiple interviews aligned with the central premise of this study, which used an iterative user-centered design approach to ensure that we were meeting the needs of our end users.

Patients had the option to complete the interviews remotely with a trained research assistant (RA) using the secure Webex conferencing platform (Cisco Systems, Inc) or via a self-administered REDCap (Research Electronic Data Capture; Vanderbilt University) form. Each interview conducted by the RA lasted 20 to 30 minutes. The guide developed for the interviews at the 3-, 6-, and 9-month study visits explored (1) patients’ experiences using i-Matter; (2) the impact (if any) that i-Matter had on patients’ motivation to participate in the study and their attitudes about diabetes, as well as on changes to self-management behaviors; and (3) recommended changes to i-Matter to improve the tool’s utility for diabetes management. In addition, questions at the 12-month visit asked about (1) the likelihood that patients would sign up for i-Matter in the future, and (2) the behaviors (if any) that patients would continue to practice now that their participation in i-Matter had ended. As compensation for participating in the i-Matter trial, patients received a US $10 gift card for completion of the 3-, 6-, and 9-month study visits. At the final study visit, patients received a US $30 gift card.

### Analysis

The interviews completed with the RA were audio recorded, translated where necessary, and transcribed verbatim. Those completed via REDCap were downloaded and formatted for analysis. The coding team consisted of the senior author (AS), a professor of population health with expertise in qualitative methods, and a research coordinator (CM) with a master’s degree in public health with qualitative research experience. Neither of the coders was associated with the participating practices. Dedoose software was used to establish, manage, and evaluate the data [18]. All data were analyzed using conventional qualitative content analysis following the process outlined in the studies by Erlingsson and Brysiewicz [19] and Hsieh and Shannon [20]. Specifically, the coders first independently read each patient’s 3-, 6-, 9-, and 12-month interview texts and identified the themes according to the study objectives. The coders then met to discuss the coding scheme and resolve any discrepancies through discussion. After this discussion, the coders reviewed the interview texts a second time to refine the overarching themes and organize the text according to the subthemes underlying each main theme.

Through this process, the study team identified 4 themes and 6 subthemes. These themes focused on reasons for participant engagement in the program, change in perception regarding healthy behaviors, sustaining healthier lifestyles, and recommendations for improving the overall patient experience with i-Matter. A final round of review and discussion was completed to discuss the subthemes and ensure that there were no inconsistencies in coding between the coders.

When interpreting the data, factors external to the study were considered, such as scientific or therapeutic developments that could have an impact on the safety of the participants or on the ethics of conducting the study.

## Results

### Patient Characteristics

Our sample comprised 71 patients, of whom 67 (94%) completed at least one interview during their participation in the intervention; 3 (4%) patients did not complete the survey-based interview, and 1 (1%) patient was unreachable for the telephone interview. Of these 67 patients, 19 (28%) were interviewed via telephone by the RA, whereas 48 (72%) completed the interviews via the REDCap survey. There were no significant differences in patient age (REDCap survey: mean 56.90, SD 8.36 years vs telephone interview: mean 57.90, SD 8.51 years; *P*=.76), sex (REDCap survey: 32/48, 66.7% female patients vs telephone interview: 10/19, 52.6% female patients; *P*=.71), or race and ethnicity (REDCap survey: 28/48, 58.3% patients of color vs telephone interview: 11/19, 57.9% patients of color; *P*=.56) between participants who completed the interview via telephone and those who completed the interview via the REDCap survey. Of the 67 patients, 67 (100%) completed the 3-month interview, 61 (91%) completed the 6-month interview, 48 (72%) completed the 9-month interview, and 67 (100%) completed the 12-month interview. Approximately half of the patients (34/67, 51%) completed interviews at all 4 time points. Table 1 displays the patient characteristics of those enrolled in the i-Matter program.

**Table 1:** Patient characteristics (n=71).

Characteristic		Values
Age (years), mean (SD)		56.65 (9.79)
Sex (female), n (%)		48 (68)
**Ethnicity, n (%)**		
	Hispanic	14 (20)
	Non-Hispanic	57 (80)
**Race, n (%)**		
	African American or Black	25 (35)
	Asian	2 (3)
	White	24 (34)
	Other	4 (6)
	Unknown	16 (23)
**Primary language, n (%)**		
	English	67 (94)
	Spanish	4 (6)
**Marital status, n (%)**		
	Single	24 (34)
	Married	29 (41)
	Divorced or separated	13 (18)
	Widowed	4 (6)
	Unknown	1 (1)
**Education, n (%)**		
	Elementary school	4 (6)
	High school	8 (11)
	Some college	16 (23)
	Associate’s degree	4 (6)
	Bachelor’s degree	16 (23)
	Graduate degree	19 (27)
	Technical school	3 (4)
	Unknown	1 (1)
Unemployed, n (%)		27 (38)
**Annual income (US $), n (%)**		
	<10,000	7 (10)
	10,000-19,999	5 (7)
	20,000-39,999	9 (13)
	40,000-59,999	17 (24)
	60,000-100,000	11 (16)
	>100,000	17 (24)
	Unknown	5 (7)
**Insurance status, n (%)**		
	None	3 (4)
	HMO^a^ or private	32 (45)
	Medicare	8 (11)
	Medicaid	11 (15)
	Medicaid or Medicare	8 (11)
	Private or Medicare	7 (10)
	State sponsored	2 (3)
Baseline HbA_1c_^b^ level, mean (SD)		8.52 (1.44)

^a^HMO: health maintenance organization.

^b^HbA_1c_: glycated hemoglobin.

### Themes

#### Overview

**Figure 2:**
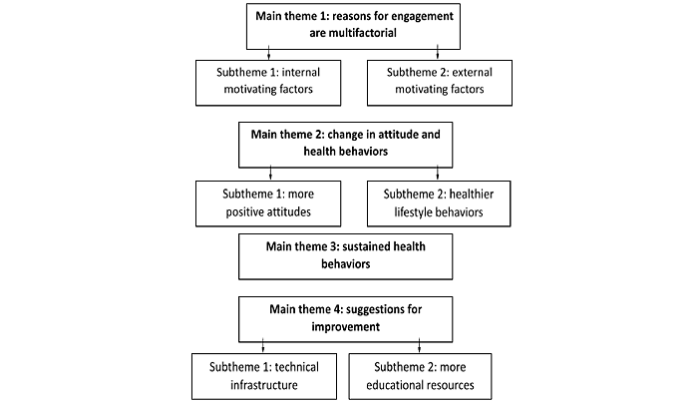
Qualitative themes and subthemes from patient data.

The themes identified in the interviews are displayed in Figure 2. The findings were characterized by 4 overarching themes, which were further broken down into 6 subthemes that support the main theme. In the following subsections, we describe each of the themes and subthemes and include exemplar quotes to support the themes. For each quote, we include the sex, age, and race and ethnicity of the participant. We also note the period during which the quote was collected (at 3, 6, 9, or 12 months).

#### Overarching Theme 1: Reasons for Engagement With i-Matter Are Multifactorial

Interview questions that elicited patients’ motivations to participate in i-Matter identified two major subthemes: (1) patients were driven by internal motivating factors that bolstered their engagement and helped them feel accountable for their diabetes and (2) external motivating factors prompted by receiving the PRO messages and personalized reports helped to serve as reminders to be consistent with their self-management behaviors.

##### Subtheme 1: Internal Motivating Factors

Internal motivating factors played a role in the patients’ engagement with i-Matter, which also affected how they felt about their health behaviors. By receiving the PRO messages, patients felt increased accountability for their health behaviors and were internally motivated to change; for example, a patient stated as follows:

It is a reminder that there are reasons to keep myself on track even when I don’t particularly feel like it.

Male patient, 34 years, White; 6-month follow-up

One patient spoke about using i-Matter and the PRO messages functioning as an accountability buddy:

[T]he program acts as an accountability buddy for anyone new to tracking sleep, eating, exercising, [and] medication.

Female patient, 52 years, White; 6-month follow-up

The greater sense of accountability translated into being more consistent with diabetes management, as stated by a patient:

Prior to the program, I rarely checked my sugar and when I did, my sugar was often higher than my target. Lately, I’m more consistently hitting my targets.

Female patient, 44 years, Hispanic; 3-month interview

In addition, patients appreciated the PRO messages because they served as an internal motivator to gain greater control over their health:

What I like most about the program is that I feel like I am being watched and have to take some responsibility for my behavior.

Male patient, 54 years, African American; 6-month interview

Increased awareness of diabetes was a second internal motivating factor that played a key role in patients’ motivation to continue seeking support for their diabetes management and contributed to their overall engagement with i-Matter. A patient stated as follows:

The program is really helping me to become more aware of my diabetes.

Female patient, 74 years, White; 9-month interview

Another individual observed as follows:

The program keeps me on track. It makes me more aware of my daily actions.

Female patient, 51 years, Hispanic; 3-month interview

Patients also discussed how the increased awareness of their diabetes as a result of the personalized reports has increased their appreciation for making healthier lifestyle choices, as a patient explained:

I’ve used the information on my report to keep me mindful on my eating habits, and physical activities, and weight loss.

Male patient, 54 years, African American; 9-month interview

Another patient agreed regarding the impact the reports had on their behaviors:

I have become more aware of the things. [Looking at the reports] I have a better understanding of the importance of doing physical activities. My A_1c_ has gone down.

Female patient, 53 years, Hispanic; 9-month interview

Another patient remarked about the awareness gained from the program at the end of the i-Matter intervention:

[I]t has made me a bit more conscious of my eating, exercise, and medication habits.

Female patient, 60 years, White; 12-month interview

##### Subtheme 2: External Motivating Factors

Several patients stated that the PRO messages they received about their diabetes served as external prompts that helped them remember to engage in healthy self-management behaviors, with a patient stating as follows:

I like that this program gives you a gentle push every day to stay on top of your health.

Female patient, 53 years, African American; 9-month interview

Several other patients echoed this feeling, especially in relation to the medication PRO message:

The daily reminders to take my medication, especially at dinnertime.

Male patient, 64 years, White; 3-month interview

My favorite is “Did you take all your diabetes medication?” There have been moments when I would have forgotten to take my medication if it wasn’t for the reminder.

Female patient, 51 years, Hispanic; 3-month interview

This patient continued to speak about the positive impacts of having the personalized report as a visual cue to develop greater consistency in managing their diabetes in later interviews:

I was able to reduce my A_1c_ from 12 to 7.8. Thank you!!! Seeing the report as opposed to just mentally thinking it’s not a big deal if I missed a day. Not realizing that one day can add up if I don’t keep track of it. Missing days of medication or not checking my sugar levels that day. I now know that each day gives me a bigger result.

Female patient, 51 years, Hispanic; 6-month follow-up

#### Overarching Theme 2: Patients’ Attitudes and Health Behaviors Changed as a Result of Participating in i-Matter

Interview questions that inquired about how participation in i-Matter changed the way patients thought about their diabetes identified two major subthemes: (1) patients developed more positive attitudes about their condition and their ability to effectively self-manage it, and (2) patients developed a better awareness of their current behaviors and were motivated to adopt healthier lifestyle behaviors.

##### Subtheme 1: More Positive Attitudes

More positive attitudes (ie, mood, feelings, and emotions) about one’s ability to manage diabetes contributed to a positive outlook about adopting healthy behaviors. These attitudes took the form of developing a more positive outlook (ie, being optimistic) about their disease, changing their perspective about their ability to control their disease outcome, and developing greater introspection about how their current behaviors relate to their disease status; for example, patients expressed optimism when discussing the impact that i-Matter had on their mindset, as a patient noted:

This program has provided hope for improving my diabetic care tremendously!

Female patient, 60 years, African American; 3-month follow-up

Another patient discussed how participation in i-Matter shifted their mindset to focus on how they could control their diabetes:

I think differently about managing my diabetes. It’s all about what you’re willing to do. Changes are always necessary, and we have to adjust our time to include managing our illness. I’m getting a little better at that—so THANK YOU!

Female patient, 50 years, White; 9-month follow-up

The shift in perspective was bolstered by the tools offered by i-Matter to help patients feel in control of their health behaviors to improve their diabetes outcomes, with a patient stating as follows:

Before, it was a death sentence, I didn’t believe a lifestyle change was possible. I look at food a little differently. I’m happy with the weight loss I obtained. Looking forward to seeing more progress.

Male patient, 50 years, African American; 9-month interview

##### Subtheme 2: Healthier Lifestyle Behaviors

Overall, patients reported positive experiences regarding participating in i-Matter, and, as a result, several of their health behaviors improved, including diet (eg, reducing carbohydrate intake or making healthier eating choices), physical activity, stress management (eg, yoga and meditation), sleep quality, weight loss, and overall control of their health; for example, a patient stated as follows:

I try to walk every day for as long as I can and try to limit my use of sweets and carbs such as bread, which I love. I take my medication because I know how important it is.

Female patient, 67 years, White; 6-month interview

This feeling was echoed by another patient who stated as follows:

I have gotten better as it is almost an alarm clock when the messages come, and I tell myself I have to get going with exercise and everything.

Male patient, 35 years, White; 6-month interview

Finally, a patient discussed the impact that i-Matter had on all aspects of their diabetes management:

It keeps me on track. It makes [me] more aware of my daily actions. I look to fit in my 30 min walk throughout the day. I am choosing healthier foods and taking my medication regularly. My blood sugar has been in the normal range.

Female patient, 51 years, Hispanic; 3-month follow-up

#### Overarching Theme 3: Patients Are Motivated to Sustain the Healthy Behaviors Adopted While Participating in i-Matter

At the 12-month interview, the questions asked patients to reflect on their participation in i-Matter and how it would affect their diabetes self-management at the conclusion of the program. Overall, patients spoke about a long-term commitment to self-managing their condition, with many stating that they would continue to engage in the healthy behaviors they had adopted while participating in i-Matter. Patients commented that the primary benefits of participating in i-Matter included an increased awareness of their daily behaviors, an understanding of the importance of staying on top of their behaviors (being consistent), and knowledge of the actions they can take to control their diabetes. A patient stated as follows:

It became a habit in making sure that I take my medication, eat well, and maintain some form of exercise. Every day. I’m in charge of my health so there are no excuses.

Female patient, 53 years, African American; 12-month interview

Patients also spoke about the willingness to continue to participate in i-Matter if it was ever offered again, with a patient stating as follows:

I would use the program for the rest of my life.

Female patient, 59 years, African American; 12-month interview

Similarly, another patient offered the following comment:

I believe that once the i-Matter program has completed, the individual participating in the program should be given the option to continue to receive text messages as prompt...in support of helping them maintain a healthier lifestyle...so the positive reinforcement could continue, even as the data collection stopped.

Male patient, 45 years, White; 12-month interview

#### Overarching Theme 4: Improvements to i-Matter Could Enhance the Patient Experience

##### Overview

Although patients spoke of the many benefits of participating in i-Matter, the tool also had shortcomings that detracted from the patient experience. Nearly half of the participants (27/67, 40%) recommended making changes to i-Matter. The subthemes identified two major areas for improvement: (1) the technical infrastructure in the design of i-Matter and (2) the need for more resources to support the data collected and shared through the tool.

##### Subtheme 1: Technical Infrastructure

A common weakness of i-Matter was the limited 2-hour time window provided to respond to PRO messages, as noted by a patient:

If I miss the text for an hour or two, then I can’t respond.

Male patient, 64 years, White; 3-month interview

Another patient echoed this point:

I do not like the timing of the messages.

Female patient, 63 years, Hispanic; 3-month interview

Patients also felt that they were missing important information in their tracking of their health behaviors because they would miss questions owing to the restrictions:

The text component is so that you cannot move to the next question until the previous one is answered. Information is being missed when the window to respond times out.

Female patient, 52 years, White; 6-month interview

Constructive feedback was also given about how to improve i-Matter. Two-thirds of the patients (44/67, 66%) recommended removing the 2-hour window so that individuals can answer whenever they are able to (or, at a minimum, have a 4-hour window). A patient provided the following feedback on the timing of the PRO messages:

Allow more time for responses as it’s not always possible to respond within the 2-hour time frame, especially when I’m traveling. Also, I don’t take my insulin until before bed, as directed, so when I’m asked if I took all my medicine by 8 PM, I can’t honestly answer yes.

Female patient, 44 years, Hispanic; 3-month interview

Of the 67 patients, 14 (21%) also commented on the number of messages. Some of the patients (5/67, 7%) felt that the PRO messages were a bit intrusive, and they were unhappy about the frequency of messages, with a patient observing as follows:

Too many messages and sometimes they come at different times of the day. It should just be one message.

Male patient, 48 years, Hispanic; 6-month interview

Another patient made the following suggestion with regard to the number of PRO messages sent per week:

Get an alternative for patients that do not have an unlimited data plan.

Female patient, 63 years, Hispanic; 12-month interview

In addition to feedback regarding the PRO message timing and frequency, a critique of the design of the survey questions as too general was offered by a patient who preferred “more precise and specific questions” (female patient, 74 years, White; 3-month follow-up). Patients also recommended greater personalization of the PRO messages, allowing the content to change over time based on the individual’s needs, including the ability to track additional optional PROs (eg, morning and evening blood glucose levels and weight) and the ability to modify answers; for example, a patient commented as follows:

Don’t use the same questions all the time. Push for people to challenge themselves and to think more about their health.

Female patient, 53 years, African American; 12-month interview

The personalized report provided was viewed as useful by a majority of the patients (58/67, 87%) to help them see the areas where they can improve; however, the design flaws limited its success, as noted by a patient:

I haven’t been able to use the information on the report to manage my diabetes because I cannot read it. Please make it viewable.

Female patient, 66 years, African American; 6-month interview

##### Subtheme 2: More Educational Resources

Nearly one-quarter (16/67, 24%) of the patients also expressed the need for more education on the topic of diabetes management (eg, soaking their feet, exercise regimens, and healthy recipes); for example, a patient commented as follows:

I would like more information sent to me on how to control my type 2 diabetes. I usually google information, but it would be nice to get information weekly.

Male patient, 54 years, African American; 3-month interview

Another patient also suggested including more dietary resources:

Ten best things to eat, in season. Ten worst things to eat regularly.

Female patient, 61 years, White; 12-month interview

Patients also recommended including education that helps to link the PROs they are tracking to diabetes outcomes; for instance, a patient stated as follows:

Maybe more education on the importance of sleeping can affect diabetes and why do we have to soak our feet.

Female patient, 42 years, Hispanic; 9-month interview

## Discussion

### Principal Findings

In this qualitative study, we sought to elucidate the ways through which our mHealth PRO tool can facilitate changes in patients’ attitudes and behaviors for improved diabetes management, as well as suggestions for improvement. Throughout their participation, patients reported a positive experience with i-Matter, which was shaped by both internal and external factors. Participating in i-Matter helped patients gain increased awareness of their condition and develop positive attitudes toward diabetes, as well as empowered them to actively take control of their condition. The increased sense of accountability created by i-Matter prompted patients to engage in self-management behaviors to improve their health. External factors, such as the PRO SMS text messages, also served as reminders to engage in healthy behaviors. Importantly, patients reported commitment to engaging in healthy behaviors once their participation in i-Matter ended. Although patients would no longer receive the PRO messages or the reports, they felt that i-Matter helped them develop new habits that they could maintain in the long term. Despite these benefits, the technical infrastructure of i-Matter was viewed as a shortcoming that potentially limited its impact. Patients also desired more educational resources to augment what they learned through tracking their behaviors via the PRO messages and reports.

Previous qualitative studies have reported similar themes related to the impact of mHealth diabetes tools on increased patient engagement and adoption of lifestyle behaviors. Qualitative analysis of the focus group data in the DiabeText study noted the benefits of an SMS text message intervention for multiple positive behavior changes, including medication adherence, diet, and physical activity [21]. Patients who participated in a digital lifestyle intervention for diabetes self-management that is similar to i-Matter also discussed the importance of increased self-awareness and reminders for sustained engagement with mHealth tools [22]. Finally, the use of the mHealth app Young with Diabetes resulted in participants feeling more connected to one another, and participation in the intervention was viewed as a positive adjunct to treatment for type 1 diabetes [23]. Similar to those who participated in i-Matter, participants in an mHealth diabetes intervention (a mobile app for persons with type 2 diabetes) recommended improvements in the functionality of the app to enhance the user experience and accommodate participant preference for more diabetes education to accompany the tool’s content [24].

Although previous studies noted an association among participation in an mHealth diabetes intervention, better patient engagement, and health behavior change, few studies [22,24] have identified the factors that underlie this relationship. A strength of our study was the ability to isolate the internal and external factors that prompted patients to change their views about their disease, become more engaged with the intervention and their health, and adopt healthy behaviors. These behavioral mechanisms (eg, perceived capability, control, and accountability), which are grounded in our theoretical model, provide important insights to drive future development of mHealth interventions that could lead to sustained behavior change. Our ongoing clinical trial will evaluate whether patients’ qualitative reports of behavior change as a result of participating in i-Matter translate into a reduction in HbA_1c_ levels and a change in self-management behaviors assessed through validated self-reported measures.

There were also some limitations to this study. First, individuals who participate in research may differ from the general population. Thus, the themes identified in this study warrant future exploration in a pragmatic trial. Second, although our intervention was cocreated with patients with type 2 diabetes, we may have missed important psychosocial factors (eg, distress) that may be relevant to this population and would serve as important mechanisms for behavior change. Third, to provide patients greater flexibility in responding to questions, we offered the option to answer the interview questions via a REDCap survey. Because of the lack of probing and other conversational techniques inherent to interviews, we may have missed important opportunities to capture patients’ nuanced experiences with i-Matter in the answers provided in the REDCap form. However, incorporating the interview questions into the battery of survey measures also increased our response rate because this methodology was viewed as more convenient for our participants because they could complete the questions in their own time. In addition, despite the inclusion of targeted questions asking about the individual intervention components in the interviews and REDCap survey, many patients talked about i-Matter as a holistic program. Thus, unless explicitly stated, the unique contribution of the PRO messages cannot be distinguished from that of the personalized reports in the patients’ responses. Future quantitative analyses will examine engagement rates with the different components to try to address this limitation. Finally, although our study is designed to capture a diverse population of patients, our sample spoke English primarily and had high educational status as well as private insurance. Thus, we may be missing important insights from populations consisting of minority groups who are disproportionately affected by type 2 diabetes [25].

### Conclusions

In conclusion, this qualitative study identified several behavioral mechanisms that can help explain the association between participation in an mHealth PRO intervention and changes in patients’ attitudes and behaviors for improved diabetes management. These mechanisms included external factors, such as reminders that served as prompts to engage in healthy behaviors, as well as internal factors that increased patients’ awareness of, and positive attitudes toward, their type 2 diabetes, as well as helped patients to feel in control of their condition, be accountable for their behaviors, and stay on top of their overall health. Addressing the limitations related to the technical infrastructure and limited educational resources of the tool may enhance its impact.

## Abbreviations

EHR: electronic health record
HbA_1c_: glycated hemoglobin
mHealth: mobile health
PRO: patient-reported outcome
RA: research assistant
REDCap: Research Electronic Data Capture

## Data Availability

The final research data from the project will not contain any patient identifiers. Research data that document, support, and validate research findings will be made available after the primary findings from the final research data set have been accepted for publication. At that time, our deidentified data will be deposited in a National Institutes of Health–supported generalist repository such as Microsoft Dataverse or Mendeley Data. To increase the findability and usefulness of the data, they will also be described with rich metadata in the NYU Data Catalog.

## References

[1] Diabetes and prediabetes. Centers for Disease Control and Prevention.

[2] The facts, stats, and impacts of diabetes. Centers for Disease Control and Prevention.

[3] American Diabetes Association (2022). Abridged for primary care providers. Clin Diabetes.

[4] Fang M, Wang D, Coresh J, Selvin E (2021). Trends in diabetes treatment and control in U.S. adults, 1999–2018. N Engl J Med.

[5] van Smoorenburg AN, Hertroijs DF, Dekkers T, Elissen AM, Melles M (2019). Patients' perspective on self-management: type 2 diabetes in daily life. BMC Health Serv Res.

[6] Sibounheuang P, Olson PS, Kittiboonyakun P (2020). Patients' and healthcare providers' perspectives on diabetes management: a systematic review of qualitative studies. Res Social Adm Pharm.

[7] Lee J, Lee E-H, Chae D, Kim C-J (2020). Patient-reported outcome measures for diabetes self-care: a systematic review of measurement properties. Int J Nurs Stud.

[8] Druce KL, Dixon WG, McBeth J (2019). Maximizing engagement in mobile health studies: lessons learned and future directions. Rheum Dis Clin North Am.

[9] Kitsiou S, Paré G, Jaana M, Gerber B (2017). Effectiveness of mHealth interventions for patients with diabetes: an overview of systematic reviews. PLoS One.

[10] Eberle C, Löhnert M, Stichling S (2021). Effectiveness of disease-specific mHealth apps in patients with diabetes mellitus: scoping review. JMIR Mhealth Uhealth.

[11] Hoppe CD, Cade JE, Carter M (2017). An evaluation of diabetes targeted apps for Android smartphone in relation to behaviour change techniques. J Hum Nutr Diet.

[12] Jimenez G, Lum E, Car J (2019). Examining diabetes management apps recommended from a Google search: content analysis. JMIR Mhealth Uhealth.

[13] Rubin R (2018). Phone apps for patients with diabetes. JAMA.

[14] Munn Z, Peters MD, Stern C, Tufanaru C, McArthur A, Aromataris E (2018). Systematic review or scoping review? Guidance for authors when choosing between a systematic or scoping review approach. BMC Med Res Methodol.

[15] Lauffenburger JC, Barlev RA, Sears ES, Keller PA, McDonnell ME, Yom-Tov E, Fontanet CP, Hanken K, Haff N, Choudhry NK (2021). Preferences for mHealth technology and text messaging communication in patients with type 2 diabetes: qualitative interview study. J Med Internet Res.

[16] Schoenthaler A, Cruz J, Payano L, Rosado M, Labbe K, Johnson C, Gonzalez J, Patxot M, Patel S, Leven E, Mann D (2020). Investigation of a mobile health texting tool for embedding patient-reported data into diabetes management (i-Matter): development and usability study. JMIR Form Res.

[17] Bradway M, Leibowitz K, Garrison KA, Howe L, Årsand E (2020). Qualitative evaluations of mHealth interventions: current gaps and future directions. Stud Health Technol Inform.

[18] Dedoose.

[19] Erlingsson C, Brysiewicz P (2017). A hands-on guide to doing content analysis. Afr J Emerg Med.

[20] Hsieh H-F, Shannon SE (2005). Three approaches to qualitative content analysis. Qual Health Res.

[21] Zamanillo-Campos R, Serrano-Ripoll MJ, Taltavull-Aparicio JM, Gervilla-García E, Ripoll J, Fiol-deRoque MA, Boylan A-M, Ricci-Cabello I (2022). Patients' views on the design of DiabeText, a new mHealth intervention to improve adherence to oral antidiabetes medication in Spain: a qualitative study. Int J Environ Res Public Health.

[22] Whelan ME, Denton F, Bourne CL, Kingsnorth AP, Sherar LB, Orme MW, Esliger DW (2021). A digital lifestyle behaviour change intervention for the prevention of type 2 diabetes: a qualitative study exploring intuitive engagement with real-time glucose and physical activity feedback. BMC Public Health.

[23] Husted GR, Weis J, Teilmann G, Castensøe-Seidenfaden P (2018). Exploring the influence of a smartphone app (Young with Diabetes) on young people's self-management: qualitative study. JMIR Mhealth Uhealth.

[24] Torbjørnsen A, Ribu L, Rønnevig M, Grøttland A, Helseth S (2019). Users' acceptability of a mobile application for persons with type 2 diabetes: a qualitative study. BMC Health Serv Res.

[25] Haw JS, Shah M, Turbow S, Egeolu M, Umpierrez G (2021). Diabetes complications in racial and ethnic minority populations in the USA. Curr Diab Rep.

